# Incremental Prognostic Value of Coagulopathy in Addition to the Crash Score in Traumatic Brain Injury Patients

**DOI:** 10.1007/s12028-020-00991-7

**Published:** 2020-05-22

**Authors:** Davi J. Fontoura Solla, Robson Luis Oliveira de Amorim, Angelos G. Kolias, Peter J. Hutchinson, Almir Ferreira de Andrade, Manoel Jacobsen Teixeira, Wellingson Silva Paiva

**Affiliations:** 1 Division of Neurosurgery, Department of Neurology, Hospital das Clínicas da Faculdade de Medicina, University of São Paulo, Dr. Enéas Carvalho de Aguiar Avenue, 255, São Paulo, 05403-000 Brazil; 2 Division of Neurosurgery, Department of Clinical Neurosciences, Addenbrooke’s Hospital, University of Cambridge, Cambridge, UK; 3 NIHR Global Health Research Group on Neurotrauma, University of Cambridge, Cambridge, UK

**Keywords:** Traumatic brain injury, Coagulopathy, Prognosis, Prognostic score, Mortality

## Abstract

**Background/objective:**

Multivariable prognostic scores play an important role for clinical decision-making, information giving to patients/relatives, benchmarking and guiding clinical trial design. Coagulopathy has been implicated on trauma and critical care outcomes, but few studies have evaluated its role on traumatic brain injury (TBI) outcomes. Our objective was to verify the incremental prognostic value of routine coagulopathy parameters in addition to the CRASH-CT score to predict 14-day mortality in TBI patients.

**Methods:**

This is a prospective cohort of consecutive TBI patients admitted to a tertiary university hospital Trauma intensive care unit (ICU) from March/2012 to January/2015. The prognostic performance of the coagulation parameters platelet count, prothrombin time (international normalized ratio, INR) and activated partial thromboplastin time (aPTT) ratio was assessed through logistic regression adjusted for the original CRASH-CT score. A new model, CRASH-CT-Coag, was created and its calibration (Brier scores and Hosmer–Lemeshow (H–L) test), discrimination [area under the receiver operating characteristic curve (AUC-ROC) and the integrated discrimination improvement (IDI)] and clinical utility (net reclassification index) were compared to the original CRASH-CT score.

**Results:**

A total 517 patients were included (median age 39 years, 85.1% male, median admission glasgow coma scale 8, neurosurgery on 44.9%). The 14-day mortality observed and predicted by the original CRASH-CT was 22.8% and 26.2%, respectively. Platelet count < 100,000/mm^3^, INR > 1.2 and aPTT ratio > 1.2 were present on 11.3%, 65.0% and 27.2%, respectively, (at least one of these was altered on 70.6%). All three variables maintained statistical significance after adjustment for the CRASH-CT score. The CRASH-CT-Coag score outperformed the original score on calibration (brier scores 0.122 ± 0.216 vs 0.132 ± 0.202, mean difference 0.010, 95% CI 0.005–0.019, *p* = 0.036, respectively) and discrimination (AUC-ROC 0.854 ± 0.020 vs 0.813 ± 0.024, *p* = 0.014; IDI 5.0%, 95% CI 1.3–11.0%). Both scores showed the satisfactory H–L test results. The net reclassification index favored the new model. Considering the strata of low (< 10%), moderate (10–30%) and high (> 30%) risk of death, the CRASH-CT-Coag model yielded a global net correct reclassification of 22.9% (95% CI 3.8–43.4%).

**Conclusions:**

The addition of early markers of coagulopathy—platelet count, INR and aPTT ratio—to the CRASH-CT score increased its accuracy. Additional studies are required to externally validate this finding and further investigate the coagulopathy role on TBI outcomes.

## Introduction

Multivariable prognostic scores are the key not only for therapeutic management and clinical decision-making, but also to reliably inform patients and relatives, to guide trials design and for benchmarking the quality of care by comparing observed and expected outcomes [1]. The corticosteroid randomization after significant head injury (CRASH) score is one of the main validated prognostic models for traumatic brain injury (TBI) [2, 3]. These and other TBI prognostic scores have underevaluated coagulopathy markers, which have been implicated on trauma and critical care patients’ outcomes.

Few studies have evaluated the role of coagulopathy markers on TBI outcomes, and most of them have not performed an analysis adjusted for already-validated prognostic scores [4, 5]. Only one study has formally evaluated the incremental prognostic value of coagulopathy in addition to a well-grounded prognostic score [6]. Indeed, coagulopathy improved the discrimination ability of the model by increasing its area under the receiver operating characteristic curve (AUC-ROC); however, the calibration and clinical utility (net reclassification index, NRI) of the new model were not fully assessed.

Here, we aimed to verify the incremental prognostic value (discrimination, calibration and clinical utility) of routine hospital admission coagulopathy parameters (platelet count, prothrombin time and activated partial thromboplastin time) in addition to the CRASH score to predict 14-day mortality outcome in TBI patients.

## Materials and Methods

This is a prospective cohort that included consecutive TBI patients admitted to a trauma intensive care unit (ICU) of a tertiary university hospital (Hospital das Clínicas da Faculdade de Medicina da Universidade de São Paulo—HC/FMUSP) from March 2012 to January 2015. Patients under 14 years old, victims of penetrating TBI or admitted from another ICU were excluded. Chronic subdural hematomas were also excluded.

Clinical, laboratory and radiological data were registered, as well as the primary outcome, 14-day death. Clinical predictors were defined as recommended by the brain trauma foundation and similar to the CRASH study protocol, including the definitions of major extracranial injury and head computed tomography (CT) alterations [2, 7]. Imaging (head computed tomography) was reviewed by a neurosurgeon blinded to the clinical data. Laboratory variables were registered at hospital admission, thus prior to the primary outcome. The percentages of missing data were: pupil reactivity at admission 5.8%; admission Glasgow coma scale (GCS) 5.0%; extracranial lesion at admission 1.2%; and age 1.2%. Since the original CRASH-CT score exact risk probability for each patient was crucial for the study objective and analysis, we decided not to execute multiple imputation and to perform a complete-case analysis.

The study protocol was approved by the local ethics committee (Comissão de Análise de Projetos de Pesquisa—CAPPesq, HC/FMUSP, protocol number 00119/10).

### Statistical Analysis

Categorical variables were described as absolute and relative frequencies and compared through the *χ*^2^ test or the Fisher exact test, as appropriate. Continuous variables distributions were evaluated by skewness and kurtosis values as well as graphical methods. Those with normal distributions were described as means and standard deviations and compared through the independent samples Student *T* test. The ones with non-normal distributions were described as medians and quartiles and compared through the Mann–Whitney test.

The performance of the coagulation parameters as predictors of 14-day death was assessed through logistic regression analysis with adjustment for the original CRASH-CT score. Platelet count, prothrombin time (international normalized ratio, INR) and activated partial thromboplastin time (aPTT, ratio) were included as binary variables for the sake of clinical utility: Platelet count was deemed low if < 100,000/mm^3^; INR was considered altered if > 1.2; and aPTT was considered abnormal if its ratio was > 1.2. Only cases with complete data for the CRASH-CT score calculations were included in the respective models. Data regarding pre-hospital hypoxia or admission glucose was missing for almost half of the cohort, so we were not able to perform the same analysis with adjustment to the extended IMPACT model.

**Fig. 1:**
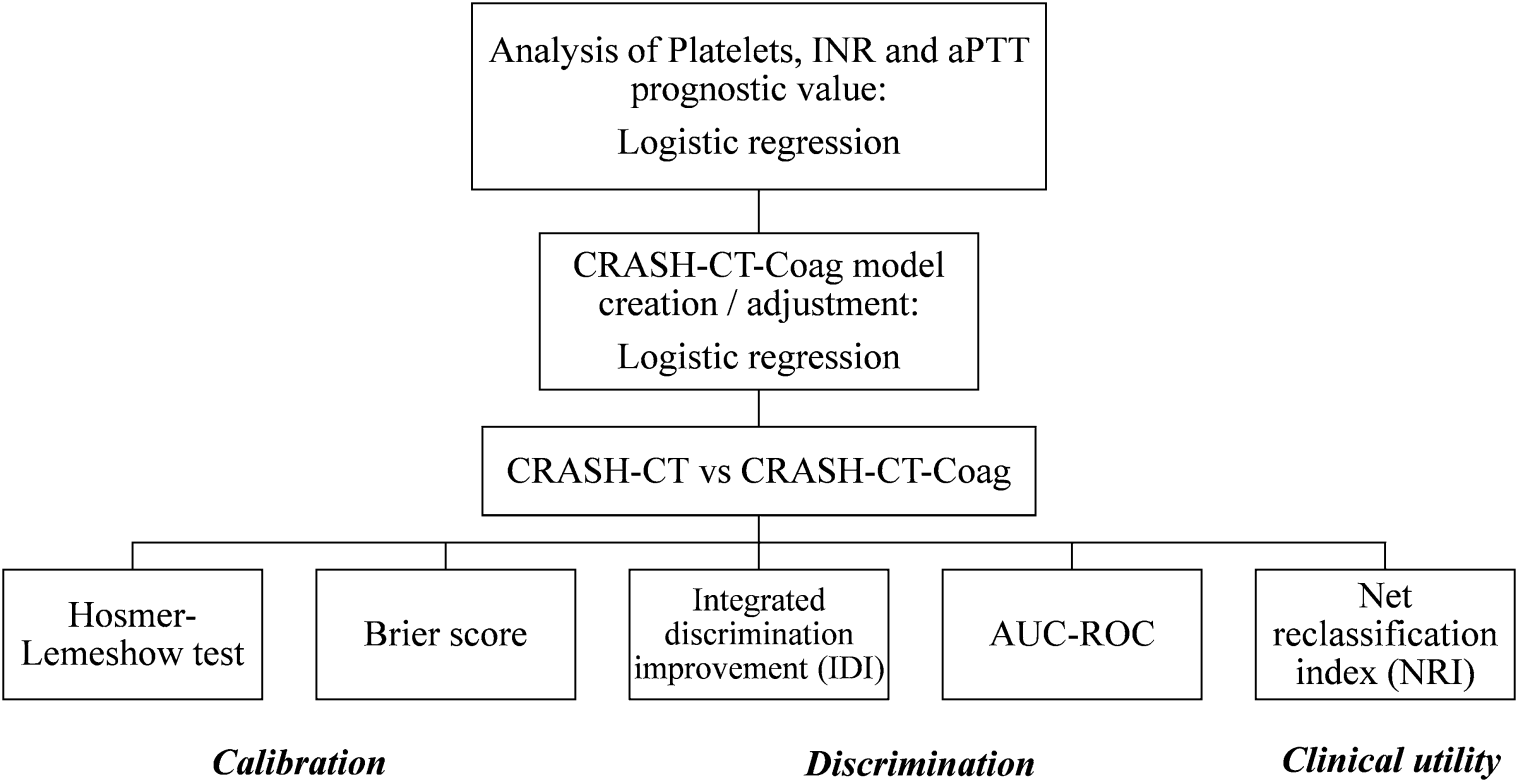
Statistical analysis flowchart

A new model, CRASH-CT-Coag, was created after the above-cited logistic regression adjustment and, thus, included the original CRASH-CT score, platelet count, INR and aPTT ratio. The latter covariates, the coagulation parameters, were included as binary variables (altered or normal). The CRASH-CT-Coag accuracy was compared to the original CRASH-CT score. The calibration was evaluated by the Hosmer–Lemeshow goodness-of-fit test, which compares the predicted and observed death probabilities in each risk decile, and by the Brier scores, a measure of the mean squared deviation between the predicted death probabilities and the observed outcomes. The discrimination was evaluated by the respective areas under the receiver operating characteristic curve (AUC-ROC), which were compared through the DeLong method, and by the integrated discrimination improvement (IDI), a measure of the difference in predicted death probabilities between the survivors and dead subjects. The clinical utility of the new prognostic model was evaluated by the net reclassification index (NRI). The NRI calculation had the assumption of the following death risk stratification: low (< 10%), moderate (10–30%) and high (> 30%) risk. The decision for this death risk stratification was empirical, but other stratification schemes were explored and generally lead to similar conclusions. Bootstrap sampling (1000 samples) was employed for the calculation of point estimates, standard errors and confidence intervals (CI). The statistical analysis flowchart is depicted in Fig. 1.

All tests were two-tailed, and final *p* values < 0.05 were considered statistically significant. These were the softwares employed for the analysis: R (R Core Team. R: A language and environment for statistical computing. R Foundation for Statistical Computing, Vienna, Austria), SPSS (IBM Corp. SPSS Statistics para Windows, versão 24.0. Armonk, NY) and MedCalc Statistical Software (MedCalc Software bvba, versão 15.2.0.0. Ostend, Belgium). The transparent reporting of a multivariable prediction model for individual prognosis or diagnosis (TRIPOD) statement was followed for this study report.

## Results

A total of 517 patients were included, with a median age of 39 (quartiles 27–52) years and 85.1% male. The three most common trauma mechanisms were fall from height (30.1%), fall from own height (26.1%) and motorcycle accident (19.5%). The median admission GCS was 8 (6–13), and pupil reactivity was absent/sluggish unilaterally on 12.9% and bilaterally on 5.9%. Major extracranial lesion was present on 54.4%, and neurosurgery was performed on 44.9% of the patients. These data and the head CT findings are summarized in Table 1. The mean 14-day death probability predicted by the original CRASH-CT score was 26.2%, and the in-hospital death probability predicted by the SAPS3 score was 26.4%. The observed 14-day and in-hospital death were 22.8% and 30.9%, respectively. The CRASH-CT score was available for 452 patients, with similar characteristics compared to the full cohort.

**Table 1:** Study sample characterization

Variables	Full cohort (517)	CRASH-CT available (452)
Age (years)	39 (27–52)	38 (26–51)
Male gender	440 (85.1)	387 (85.6)
Trauma mechanism		
Fall from height	156 (30.2)	141 (31.2)
Fall from own height	135 (26.1)	117 (25.9)
Motorcycle accident	101 (19.5)	89 (19.7)
Car accident	55 (10.6)	49 (10.8)
Other	70 (13.5)	56 (12.4)
Glasgow coma scale	8 (6–13)	8 (6–13)
Motor score	5 (4–6)	5 (4–6)
Pupil reactivity		
None	29 (6.0)	26 (5.8)
One	63 (12.9)	59 (13.1)
Both	395 (81.1)	367 (81.2)
Major extracranial lesion	278 (54.4)	255 (56.4)
Head computed tomography		
Marshall classification		
I	21 (4.1)	18 (4.0)
II	215 (41.6)	196 (43.4)
III	43 (8.3)	36 (8.0)
IV	21 (4.1)	20 (4.4)
V	211 (40.8)	178 (39.4)
VI	6 (1.2)	4 (0.9)
Hemorrhagic petechiae	70 (13.5)	62 (13.7)
Compressed III VT or basal cisterns	90 (17.4)	71 (15.7)
Traumatic SAH	465 (89.9)	403 (89.2)
Midline shift > 5 mm	153 (29.6)	128 (28.3)
Hematoma	342 (66.2)	292 (64.6)
Effaced cortical sulci	224 (43.3)	192 (42.5)
Intraventricular hemorrhage	112 (21.7)	98 (21.7)
Skull fracture	263 (50.9)	233 (51.5)
Surgically evacuated hematoma	232 (44.9)	197 (43.6)
Prognostic scores		
CRASH-CT model 14-day death probability (%)	–	26.2 ± 22.9
SAPS 3 for in-hospital death (points)	51.3 ± 15.4	51.0 ± 14.9
Mortality		
Fourteen-day	118 (22.8)	99 (21.9)
In-hospital	160 (30.9)	132 (29.2)

Categorical data are presented as *n* (%) and continuous data as median (quartiles) or mean ± standard deviation

*SAH *subarachnoid hemorrhage, *SAPS3* simplified acute physiology score 3, *VT* ventricle

Thrombocytopenia, INR > 1.2 and aPTT > 1.2 were present on 11.3%, 65.0% and 27.2%, respectively. At least one of these parameters was altered on 70.6%. The univariate analyses for the coagulation parameters are presented in Table 2. Platelet count, prothrombin time and aPTT levels were associated with 14-day outcome. Platelet count was lower, and thrombocytopenia (< 100,000/mm^3^) was more common on the subgroup that evolved to death. Similarly, INR and aPTT were higher and more commonly altered (> 1.2) on this subgroup. These results were the same for the subset with the CRASH-CT score available (data not shown).

**Table 2:** Coagulation parameters

Variables	Total (517)	14-day outcome		*p* value
		Dead (118)	Alive (399)	
Platelets × 10^5^ mm^3^	178 (137–227)	162 (115–218)	185 (147–228)	0.003
Platelets < 100,000/mm^3^	57 (11.3)	26 (22.4)	31 (8.0)	< 0.001
Prothrombin time (INR)	1.28 (1.17–1.46)	1.40 (1.25–1.67)	1.25 (1.15–1.40)	< 0.001
INR > 1.2	335 (65.0)	95 (80.5)	240 (60.5)	< 0.001
APTT	1.09 (0.97–1.22)	1.19 (1.09–1.47)	1.05 (0.95–1.18)	< 0.001
aPTT > 1.2	140 (27.2)	54 (46.2)	86 (21.6)	< 0.001

Categorical data are presented as *n* (%) and continuous data as median (quartiles)

*APTT* activated partial thromboplastin time, *INR* international normalized ratio

The three coagulation parameters of interest were included as binary variables (normal or altered) on the multivariable logistic regression analyses. Platelet count below 100,000/mm^3^ (OR 2.45, 95% CI 1.14–5.25, *p* = 0.021), INR above 1.2 (OR 2.03, 95% CI 1.05–3.94, *p* = 0.036) and aPTT ratio above 1.2 (OR 2.50, 95% CI 1.41–4.48, *p* = 0.002) maintained statistical significance after adjustment for the CRASH-CT score. More details of the multivariable model are presented in Table 3.

**Table 3:** Multivariable logistic regression model for 14-day mortality prediction

Variables	Coef	SE	OR	95% CI	*p* value
CRASH-CT adjusted model					
Platelets < 100,000/mm^3^	0.896	0.389	2.45	1.14–5.25	0.021
INR > 1.2	0.708	0.338	2.03	1.05–3.94	0.036
aPTT > 1.2	0.917	0.293	2.50	1.41–4.48	0.002

*aPTT* activated partial thromboplastin time, *CI* confidence interval, *Coef* coefficient, *INR* international normalized ratio, *OR* odds ratio, *SE* standard error

**Fig. 2:**
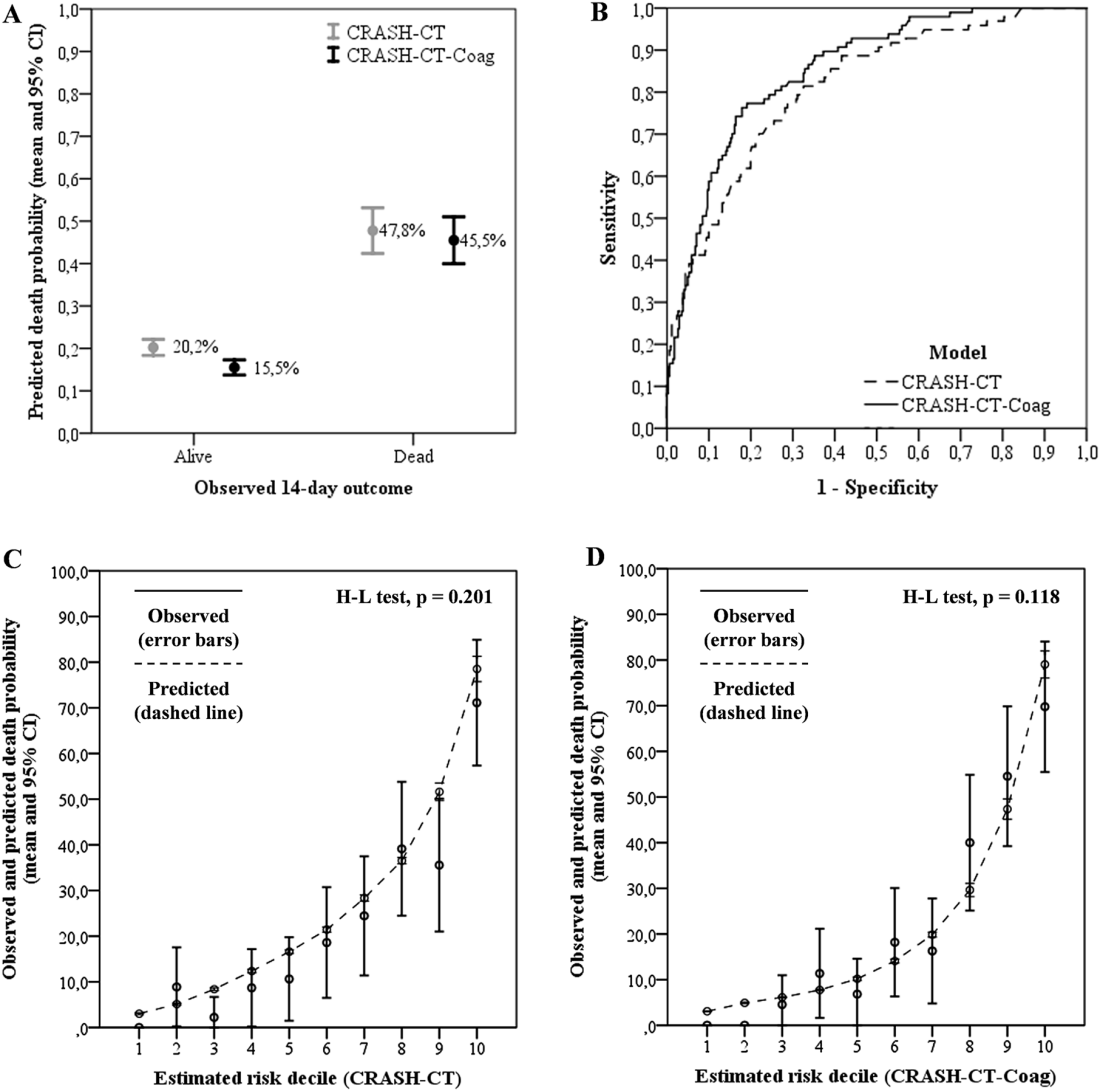
Panel A: Observed outcomes and predicted death probability according to each model. The CRASH-CT-Coag score outperformed the CRASH-CT score on calibration (Brier scores 0.122 ± 0.216 vs 0.132 ± 0.202, mean difference 0.010, 95% CI 0.005–0.019, *p* = 0.036). This indicates that the new model leads to overall smaller differences between the predicted death probabilities and the observed outcomes. Also, the mean risk difference on predicted death probabilities for those who survived and those who did not was higher for the CRASH-CT-Coag model (29.4%, 95% CI 21.1–39.0 vs 24.4%, 95% CI 16.3–33.2, *p* < 0.001). This implies an integrated discrimination improvement (IDI) of 5.0% (95% CI 1.3–11.0%), which is the additional difference on predicted death probabilities between the survivors and the dead subjects; Panel B: Receiver operating characteristic (ROC) curve analysis. The CRASH-CT-Coag score also had a better discrimination. The AUC-ROC for the new model was 0.854 ± 0.020 vs 0.813 ± 0.024 for the original one (*p* = 0.014); Panels C and D: Observed and predicted death probabilities by estimated risk deciles. Both scores showed satisfactory results by the H–L test (*x*^2^ = 12.8, *p* = 0.118 and *x*^2^ = 11.0, *p* = 0.201, respectively)

As stated on the methods section, a new model, CRASH-CT-Coag, was created and compared to the original CRASH-CT model regarding calibration, discrimination and clinical utility. The mean 14-day death probability predicted by the CRASH-CT-Coag was 22.1% (± 12.2). The CRASH-CT-Coag score outperformed the CRASH-CT score on calibration as assessed by the Brier scores (0.122 ± 0.216 vs 0.132 ± 0.202, mean difference 0.010, 95%CI 0.005–0.019, *p* = 0.036, respectively). This indicates that the new model leads to overall smaller differences between the predicted death probabilities and the observed outcomes (Fig. 2, panel A). Both scores showed the satisfactory results by the H–L test (*x*^2^ = 12.8, *p* = 0.118 and *x*^2^ = 11.0, *p* = 0.201, respectively) (Fig. 2, panels C and D).

The CRASH-CT-Coag score also had a better discrimination. The AUC-ROC for the new model was 0.854 ± 0.020 versus 0.813 ± 0.024 for the original one (*p* = 0.014) (Fig. 2, panel B). Also, the mean risk difference on predicted death probabilities for those who survived and those who did not was higher for the CRASH-CT-Coag model (29.4%, 95% CI 21.1–39.0 vs 24.4%, 95% CI 16.3–33.2, *p* < 0.001). This implies an IDI of 5.0% (95% CI 1.3–11.0%), which is the additional difference on predicted death probabilities between the survivors and the dead subjects (Fig. 2, panel A).

**Fig. 3:**
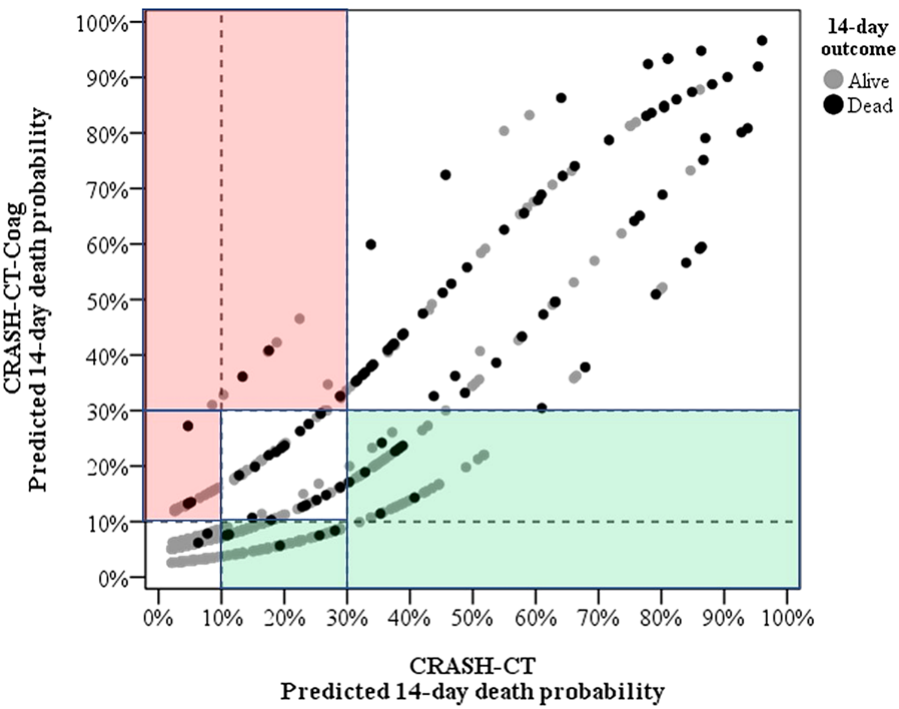
Predicted death probabilities for the original and new models according to risk strata and 14-day outcome. The new predicted death probability strata are higher on the red area, the same on the white area and lower on the green area. The four discernible groups visually seen reflect the difference between the models under evaluation, which is due to the three dichotomous variables (normal or altered platelet count, INR and aPTT ratio, which odds ratio are similarly around 2–2.5)

Considering the strata of low (< 10%), moderate (10–30%) and high (> 30%) risk of death, the CRASH-CT-Coag model, compared to the original CRASH-CT, would yield a global net correct reclassification of 22.9% (95% CI 3.8–43.4%) (Table 4). Among the survivors, the NRI to a lower risk stratum was 8.5% (95% CI 0.3–26.9%). Among the dead ones, the NRI to a higher risk stratum was 14.4% (95% CI 0.9–22.6%). In other words, the CRASH-CT-Coag score had a better performance regardless of the outcome. In Fig. 3, the predicted death probabilities by the new model were plotted against the probability predicted by the original CRASH-CT model. The new predicted death probability strata were higher on the red area, the same on the white area and lower on the green area. Thus, the ideal scenario would be for the dead subjects to be on the red area and the survivors on the green area. The four discernible groups visually shown in Fig. 3 reflect the difference between the models underevaluation, which is due to the three dichotomous variables (normal or altered platelet count, INR and aPTT ratio, which OR are similarly around 2–2.5).

**Table 4:** Stratified predicted death probability according to each model and outcomes

	CRASH-CT-Coag		
	< 10%	10–30%	> 30%
Fourteen-day outcome: dead			
CRASH-CT			
< 10%	2	3 ^a^	0^a^
10–30%	5^b^	16	3^a^
> 30%	0^b^	8^b^	60
Fourteen-day outcome: alive			
CRASH-CT			
< 10%	103	19^b^	1^b^
10–30%	84^a^	49	10^b^
> 30%	1^a^	37^a^	37

^a^Patients correctly reclassified by the CRASH-CT-Coag model

^b^Patients wrongly reclassified by the CRASH-CT-COag model

Case examples are presented in Table 5 to illustrate how the adjusted model could impact the predicted outcome probability.

**Table 5:** Case examples, predicted death probabilities by each model and observed outcomes

Model variables	Case 1	Case 4	Case 5	Case 3	Case 2	Case 6	Case 7
Age	31	66	39	80	69	46	22
Admission GCS	13	7	10	10	14	6	4
Reactive pupils	Both	One	Both	Both	Both	Both	Both
Major extracranial injury	No	Yes	Yes	Yes	Yes	No	Yes
Imaging (head CT)							
Petechial hemorrhages	No	No	No	Yes	No	No	No
Basal cisterns or III VT obliteration	No	No	Yes	No	No	No	No
Subarachnoid bleeding	Yes	Yes	Yes	Yes	Yes	Yes	Yes
Midline shift	No	No	Yes	No	No	No	No
Hematoma	Yes	Yes	Yes	Yes	Yes	No	Yes
Coagulation							
Platelets (/mm^3^)	18.000	115.000	205.000	83.000	17.000	112.000	189.000
INR	1.33	1.18	1.06	2.53	1.48	1.02	1.10
aPTT	1.50	0.96	1.10	1.23	1.90	0.80	0.97
Predicted 14-day death probability (%)							
CRASH-CT	4.7	51.7	28.1	45.7	13.3	19.3	32.0
CRASH-CT-Coag	27.2	22.0	8.4	72.4	36.1	5.7	9.9
Fourteen-day outcome	Dead	Alive	Alive	Dead	Dead	Alive	Alive

*aPTT* activated partial thromboplastin time, *CT* computed tomography, *GCS* glasgow coma scale, *INR* international normalized ratio, *VT* ventricle

## Discussion

Coagulopathy is common after TBI, and its reported incidence may vary from 10% to more than 90%, depending on the definition, for which there is no consensus yet [8, 9]. This disorder may start from minutes to hours after TBI and, although most develop it at the first 24 h, it may occur up to the fifth-day post-injury and extend for more than 72 h in 30% of the cases [10]. It seems to be associated with the severity of the injury, rather than its location [11, 12]. However, coagulopathy itself exerts an independent effect on mortality after TBI [10, 12, 13]. Thus, the hypothesis that coagulopathy markers would improve the available TBI scores is sound.

We have shown that coagulopathy markers may increase the prognostic accuracy of the CRASH-CT score, although it performed quite well in our population. Each altered parameter, platelet count, INR or aPTT ratio, more than doubled the odds of death. Different methodologies were used to assess all components of accuracy and were consistent on showing the new model superiority regarding discrimination, calibration and clinical utility. The parameters analyzed are inexpensive and usually routinely measured on trauma and intensive care patients. Also, the cutoffs used for the dichotomous classification (altered or normal) are internationally standardized and well-grounded on clinical practice—rather than artificial cutoffs to maximize statistical significance. To our knowledge, this is the one of the most comprehensive evaluations of routine coagulopathy markers for TBI prognostication. Yuan et al. [14] have also demonstrated that the addition of the coagulation test results (INR and aPTT) to a newly developed multivariable model including age, neurological examination, imaging and glucose can improve its predictive ability, but they have not compared it against a previously validated score (as the CRASH or the IMPACT models).

Some critical care scores do include some of these coagulopathy indicators as risk markers or worse outcome predictors. Moreover, although the original CRASH score did not evaluate laboratory variables, the international mission for prognosis and analysis of clinical trials in traumatic brain injury (IMPACT) original study analysis—another well-grounded TBI prognostic tool—was significant for platelets and INR as predictors of outcome [15]. Ultimately, platelets and INR were not included in the final IMPACT score because these measures were available on less than 20% and 10%, respectively, of the total study sample. The aPTT values were not analyzed in the IMPACT study.

The above relates to the first limitation to be noted in our study. The IMPACT extended model includes variables that were not fully addressed by the protocol of this study and hypoxia and glucose were not available at admission for almost half of the sample. Thus, we could not perform a similar analysis with adjustment for the IMPACT extended model. However, the IMPACT model was developed based on patients from high-income countries, whereas the CRASH data were mainly collected from low- and middle-income ones. It has been shown that the IMPACT model may not fit the CRASH data so well [16]. Even though we only analyzed the early 14-day vital status, this is a trustworthy outcome and robust against rehabilitation capacity shortages, which is still a problem in middle-income countries and impacts the long-term clinical results. Another constraint is the literature heterogeneity regarding the definition of coagulopathy. For instance, coagulopathy incidence variation has been attributed to differences in study designs, injury diversity, different time points for testing of coagulation parameters and the absence of a universally recognized definition of coagulopathy [8, 9]. As we said, the selected cutoffs for platelet count, INR and aPTT ratio are standard and common on clinical practice, but we recognize that any dichotomization of a continuous variable may carry some bias and may be criticized. The dynamic nature of TBI poses additional challenges on that matter. Lastly, although the incremental prognostic value of coagulopathy was consistent across different methods of analysis, an external validation of this proposal of prognostic model extension is crucial for its endorsement and clinical application. Additional tests for the evaluation of the coagulation system—enzymatic processes, fibrinolysis and platelets function—are becoming more widely available (e.g., fibrinogen and thromboelastography), and these may gain further relevance on coagulopathy management and outcome prognosis.

Opportunities for updating the CRASH and IMPACT models may be brought in the near future by noteworthy studies, including the Collaborative European NeuroTrauma Effectiveness Research in Traumatic Brain Injury (CENTER‐TBI) study, Transforming Research and Clinical Knowledge in TBI (TRACK‐TBI) dataset and Collaborative REsearch on ACute Traumatic Brain Injury in intensiVe Care Medicine in Europe (CREACTIVE) [17–19].

## Conclusion

The addition of platelet count, INR and aPTT, interpreted as early markers of coagulopathy, to the CRASH-CT score increased its accuracy, with better discrimination, calibration and clinical utility. Additional studies are required to externally validate this finding and further investigate its implications for TBI management. Moreover, this result underscores the need for further investigation of the coagulopathy role on TBI outcomes.

## Abbreviations

aPTT: Activated partial thromboplastin time
AUC-ROC: Area under the receiver operating characteristic curve
CENTER‐TBI: Collaborative European NeuroTrauma Effectiveness Research in Traumatic Brain Injury study
CREACTIVE: Collaborative REsearch on ACute Traumatic Brain Injury in intensiVe Care Medicine in Europe
CRASH: Corticosteroid randomization after significant head injury
CT: Computed tomography
GCS: Glasgow coma scale
HC/FMUSP: Hospital das Clínicas da Faculdade de Medicina da Universidade de São Paulo
ICU: Intensive care unit
IDI: Integrated discrimination improvement
IMPACT: International mission for prognosis and analysis of clinical trials in traumatic brain injury
INR: International normalized ratio
NRI: Net reclassification index
TBI: Traumatic brain injury
TRACK‐TBI: Transforming research and clinical knowledge in TBI
TRIPOD: Transparent reporting of a multivariable prediction model for individual prognosis or diagnosis
